# Caffeic Acid Prevents Vascular Oxidative Stress and Atherosclerosis against Atherosclerogenic Diet in Rats

**DOI:** 10.1155/2022/8913926

**Published:** 2022-01-13

**Authors:** Ying Wang, Gurpreet Kaur, Manish Kumar, Ajay Singh Kushwah, Atul Kabra, Ritu Kainth

**Affiliations:** ^1^Department of Ophthalmology and Otorhinolaryngology, Xi'an No. 3 Hospital, The Affiliated Hospital of Northwest University, Xi'an, Shaanxi 710018, China; ^2^Department of Pharmacology, Amar Shaheed Baba Ajit Singh Jujhar Singh Memorial College of Pharmacy, Bela, Ropar, Punjab, India; ^3^Chitkara College of Pharmacy, Chitkara University, Rajpura, Punjab, India; ^4^University Institute of Pharma Sciences, Chandigarh University, Gharuan, Mohali, Punjab, India

## Abstract

Diet and lifestyle play a crucial role in the progress of some cardiovascular disorders (CVDs). Rising interest in natural products and their pharmacological investigations witnessed therapeutic potential against CVDs. Caffeic acid (CA) is an organic composite hydroxycinnamic acid derivative classified among phenolics. It is a secondary metabolite biosynthesized in all plant species in the form of ester conjugates. The reported pharmacological activities of CA are neuroprotective, cardioprotective, hypoglycemic, antioxidant, and immunomodulatory properties. This work is aimed to examine the outcome of CA in atherogenic diet- (Ath-) induced rat model on lipid profile changes and endothelium function. The method involves a study duration of 35 days utilizing (*n* = 6) male Wistar rats (180–200 g) that were fed either normal chow or Ath. Study groups are given (i) normal chow diet, (ii) Ath, (iii) Ath + CA (25 or 50 mg/kg, *p.o.*), (iv) normal chow diet + CA (50 mg/kg, *p.o.*), and (v) Ath + Atorvastatin (ATORVA) (5 mg/kg, *p.o.*). Blood samples were collected at the end of the study to measure serum lipid profile, alanine aminotransferase, aspartate aminotransferase, lactate dehydrogenase, and tissue oxidative stress level. Hemodynamic parameters and aorta staining were performed. CA treatment ameliorated lipid profile and significantly reduced the oxidative stress level. Aorta staining examination revealed a marked reduction of the atherosclerotic lesions. These findings suggested that CA is an effective treatment approach for preventing atherosclerotic lesion progression attributed to protection against oxidative stress and various enzymatic activities in the Ath model.

## 1. Introduction

At present, cardiovascular origin disorders such as atherosclerotic cardiovascular disease (ASCVD) are the chief reason of illness and death across the globe [1]. In addition to well-established risk factors (e.g., hypertension, dyslipidemia, and smoking), some other factors such as choice of lifestyle, food habits, occupation, and physical inactivity complicate body homeostasis that greatly enhances the risk of ASCVD and other cardiometabolic disorders that may lead to life-threatening situations [2]. Obesity itself jeopardizes health with diminished quality of life [3]. According to an estimate by the WHO (World Health Organization), India spent $200–250 billion on healthcare (particularly CVDs) over a period of 10 years. Several reasons attributed for the higher affliction rate of CVD, fatal outcomes, and mortality are inherent pathobiological mechanisms, social factors, lifestyle, alcohol abuse (smoking), and their interactions [4]. Amongst various factors, hypercholesterolemia is directly linked with obesity and is the primary trigger of underlying inflammation, insulin resistance, and oxidative stress [5]. Higher total cholesterol (TC) levels and low-density lipoprotein cholesterol (LDL-C) help immensely foster an atherosclerotic plaque in the coronary artery. Intake of diets with high content of cholesterol and saturated fats (i.e., Western-type diets) for chronic periods are associated with the amplification of risk of CVDs. One of the most common causes of CVDs is hypercholesterolemia, and an increase in serum LDL-C and TC are the most important risk factors for the development of inflammatory insult, damage to the vessel wall, platelet activation, and subsequent progression of atherosclerosis [6]. Gathering of lipids within arterial walls is the hallmark feature in atherosclerosis that leads to fatty streak and formation of lipid-foam cells in the intima of an artery, which ultimately gets hardened and forms plaque, thereby causing artery constriction and hardening resulting in full blockage in later stages [7]. Plaque buildup in the blood vessels of the heart is responsible for the coronary artery disease, which further leads to a heart attack. Similarly, blockage in brain vessels can lead to ischemic stroke that has widespread implications associated with the death of the patient [8].

Atherosclerosis can be healed by altering lifestyle and eating habits. The treatment of atherosclerosis is centered on either lowering cholesterol synthesis or lowering the synthesis of low-density lipoproteins. At present, statins and peroxisome proliferator-activated receptor (PPAR) agonists are the most widely used medications to combat hyperlipidemia and associated cardiovascular ailments [9]. The main mode of action of statins is in the hepatic cells, where they hinder the 3-hydroxy-3-methyl-glutaryl-coenzyme A reductase (HMG-CoA reductase) enzyme, which catalyzes the rate-limiting stage in the metabolic pathway that gives rise to cholesterol and isoprenoids [10, 11]. A few other drugs such as niacin and fibrates are also used across the globe in hypercholesterolemia conditions that target the reduction of LDL-C formation [12]. However, long-term use of statins (e.g., simvastatin and rosuvastatin) has been associated with adverse effects such as dizziness, gastrointestinal complications, muscle pain, sleep problems, decrease in platelet count, hair loss, hepatitis, pancreatitis, and loss of libido (erectile dysfunction) [13]. These adverse effects along with low patient compliance to the existing hypocholesterolemia drugs propelled seeking alternative treatment approaches, which can not only ameliorate the cholesterol profile but also reduce the risk of developing cardiovascular disorders.

Recently, rejuvenated inquisitiveness in medicinal herbs and biological active dietary products have convinced preventive approaches in the therapeutics of CVDs. Considerable attention on plant phenolic compounds suggested potential evidence against many disorders such as cancer, neurodegenerative disorders, and heart diseases. Antioxidant potency and ubiquitous presence in nature render these phenolic compounds easily available, devoid of major adverse effects when consumed in the long term that enhance patient compliance many-fold [14]. Hydroxycinnamic acids are phenolic compounds with many biological effects such as anti-inflammatory [15], antiviral, antibacterial, antiatherogenic [16], and anticarcinogenic [17]. Although several derivatives of hydroxycinnamic acid are found in many plants, however, pharmacologically the most essential and common prototype of hydroxycinnamic acids is caffeic acid (CA) (3, 4-dihydroxycinnamic acid). CA occurs in fruits, olives, coffee beans, grains, propolis, and many dietary supplements [18]. Furthermore, CA has free-radical-scavenging and metal ion-chelating properties and can also fortify endogenous antioxidants that form the basis of detoxifying mechanisms in the body [19]. CA targets several signaling pathways (e.g., p38 MAPK, transcription factor and signal translation 3, metallopeptidase, nuclear factor kappa B, and adhesion molecules) and molecular mechanisms (e.g., nitric oxide, 5-lipoxygenase, calcium, potassium channels, and adrenergic receptors) associated with oxidative stress, inflammation, and immunomodulation that may modify the pathogenesis of hypercholesterolemia and CVDs [17, 20]. As CA is an antioxidant, it may shield cell components against oxidative mutilation and consequently impede the peril of numerous degenerative ailments linked with pro-oxidative sequences [21].

## 2. Methods and Materials

### 2.1. Experimental Animal and Study Protocol

**Figure 1:**
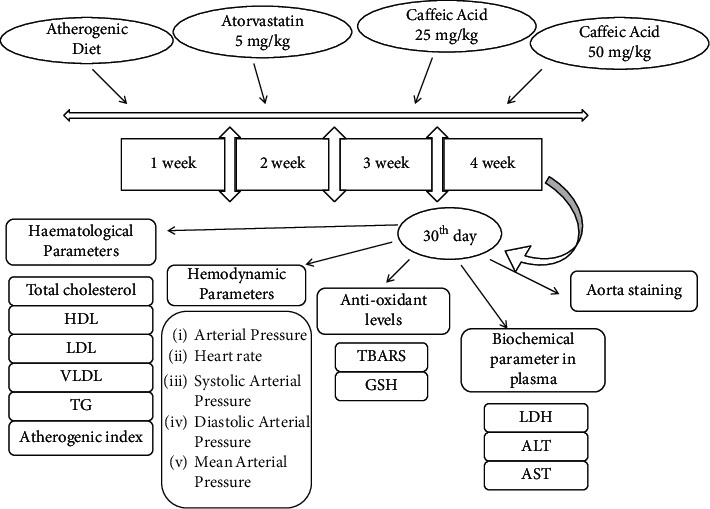
Experimental design.

Albino Wistar rats of either sex (6–8 weeks old having 180 ± 20 g body weight) were arbitrarily selected from the departmental animal facility. Standard size polypropylene cages with husk as bedding were used to house the animals under regulated temperature (23 ± 2°C), humidity (40 ± 10%), and a 12 : 12 h dark/light cycle environment using artificial lights. As per CPCSEA guiding principles, typical diet and reverse osmosis purified water was offered to the rats *ad libitum*. The research protocol had been permitted by the Institutional Animal Ethics Committee (IAEC) of Amar Shaheed Baba Ajit Singh Jujhar Singh Memorial College of Pharmacy, Bela, Ropar, Punjab (Approval no. ASCB/IAEC/08/15/101). Five rats per cage were allowed to acclimatize for one week before the experiments.  Figure 1 illustrates a schematic demonstration of the experimental design and treatment timeline. The atherosclerogenic diet (Ath) model was used to induce atherosclerosis in rats. The composition of the diet (for 1 kg) was as follows: normal chow diet (945 g), cholesterol (10 g), pig lard (25 g), multivitamins (10 g), and minerals (10 g) [22]. Thirty animals were randomly divided into six groups (*n* = 6 in each group) in a single-blind pattern: (i) control group, (ii) Ath control (atherogenic diet), (iii) Ath + CA (25 mg/kg), (iv) Ath + CA (50 mg/kg), (v) control + CA (50 mg/kg), and (vi) Ath + Atorvastatin (ATORVA) (5 mg/kg). Both test drugs, CA (25 and 50 mg/kg) [23–25], and standard drugs, Atorvastatin (5 mg/kg) [26], were administered once a day daily for 30 days *via* the intraperitoneal (*i.p.*) route. In previous studies, no major adverse effects of CA were reported at dose range (5–200 mg/kg) in rats; however, some minor side effects were apparent in pregnant female mice [27, 28]. Control and Ath control groups received an equivalent volume of drug vehicle (normal saline with dose-volume 5 ml/kg) alone for 30 consecutive days. Control and control + CA (50 mg/kg) groups were given a normal chow diet. The mean body weight of rats was analyzed on the first day and subsequently weekly monitored. Blood samples were taken on day 30, and the cervical dislocation technique under anesthesia (sodium pentobarbitone, 150 mg/kg, *i.p.*) was used to euthanize all the animals.

### 2.2. Estimation of Hemodynamic Functions

Rats were subjected to anesthesia using 25% urethane (1.5 g/kg, *i.p.*). During whole investigational procedures, body temperature of the animals was sustained at 37 ± 0.5°C using a heating pad. To perform a tracheotomy, the neck region was cut open with a ventral midline incision. The left carotid artery was cannulated using a polyethylene tube (external diameter 0.40 mm; internal diameter 0.30 mm) fastened to a 3-way cannula. The cannula was heparinized (heparin 300 IU/ml), and for the measurement of heart rate (HR), systolic (SAP), diastolic (DAP), and mean arterial pressures (MAPs), it was attached to POWER LAB 4/30 (AD Instruments, NSW, Australia) arrangement using a pressure transducer.

### 2.3. Assessment of Biomarkers in Blood Samples

After the completion of drug treatment duration, the blood samples (1.5–2 ml) were taken by piercing retro-orbital plexus of rats. Serum was isolated from blood by centrifuging (REMI, Mumbai) the samples for 10 min at room temperature with 1000 ×g force to execute biochemical tests. Separated serum diverse enzyme markers such as alanine aminotransferase (ALT/SGPT) (IU/L), aspartate aminotransferase (AST/SGOT) (IU/L), and lactate dehydrogenase (LDH) (IU/L) were quantified. Lipid profile was evaluated by quantifying total cholesterol (TC; mg/dL), HDL (high-density lipoprotein; mg/dL), LDL (low-density lipoprotein; mg/dL), VLDL (very-low-density lipoprotein; mg/dL), and triglycerides (mg/dL) levels in the blood samples [29]. Atherogenic index in plasma (AIP) was quantified using the formula AIP = (TC − HDL)/HDL. Standard techniques were followed for quantification of the enzyme activities and lipid profile as per the instructions booklet given in the kits procured from Arkray Healthcare Pvt., Ltd., Mumbai (India) (AutoSpan®) and Reckon Diagnostics P. Ltd., Vadodara (India).

### 2.4. Evaluation of Oxidative Stress Biomarkers

Immediately after blood sample collection, the rats were humanely euthanized using a cervical dislocation technique under anesthesia. The entire heart was surgically removed, pulverized to minor parts (1 cm^3^), and prepared for the preparation of 10% w/v homogenate. Subsequently, the heart sections were homogenized (REMI, Mumbai) in ice-cold phosphate-buffered saline (0.05 M PBS) and centrifuged (3000 ×g, 10 min, 4°C) to obtain the supernatant that was skimmed off for assessment of biomarkers of oxidative stress [29]. Thiobarbituric acid reactive substances (TBARS) were estimated to determine malondialdehyde levels (*μ*mol/ml), which is a key lipid peroxidation product [30]. Reduced glutathione (GSH) (nmol/ml) level was estimated to assess endogenous antioxidant levels [31]. Standard protocols were followed for estimating the biomarkers of oxidative stress.

### 2.5. Aorta Staining Method

The external wall of the aorta was organized for amputation of perivascular fat, and the aorta was stained using dye Oil Red O (ORO) as defined in previous reports [32]. After euthanasia of all the animals, the aorta was harvested and rinsed swiftly in cold water to get rid of surplus blood and tissues. The aortic section was positioned in 10% formalin solution for 10 min duration. Afterward, it was rinsed using phosphate buffer solution (pH 7.4 PBS) two or three times as required. Each section was dipped in the ORO solution for 15 min duration at room temperature. Oil Red O-stained regions were quantified using the Image Pro Plus image analysis system.

### 2.6. Statistical Analysis

Data were gathered and subsequently analyzed by an experienced researcher using a one-way ANOVA followed by Tukey's multiple comparison tests. Data were stated as mean ± standard error of the mean (S.E.M.) in this study. A value of *p* < 0.05 was deemed to be significant.

## 3. Results

### 3.1. CA Prevents Body Weight Gain in Rats Maintained on Ath

**Figure 2:**
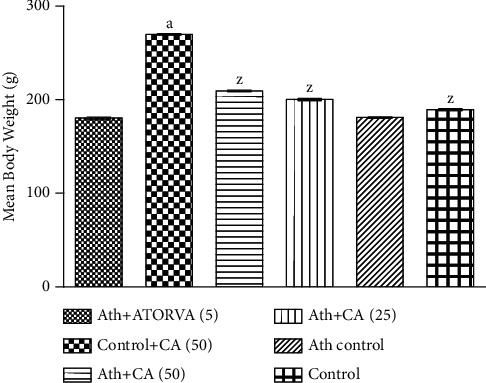
Caffeic acid (CA, 25 and 50 mg/kg) prevents body weight gain in rats exposed to atherosclerogenic diet (Ath). Values are expressed as mean ± S.E.M. ^a^*p* < 0.001*vs.* the control group; ^z^*p* < 0.001*vs.* the Ath control group.

The Ath control group exhibited a significant (*p* < 0.001) increase in mean body weight (*g*) when juxtaposed to the saline control group. Ath + CA 25 mg/kg rats showed a significant decline in the mean body weight (*p* < 0.001) relative to the Ath control group. The higher dose of CA (50 mg/kg) and standard drug (ATORVA 5 mg/kg) showed a significant (*p* < 0.001) reduction in the mean body weight of rats that were given Ath diet in comparison to rats in the Ath control group (Figure 2).

### 3.2. CA Prevents Ath-Triggered Derangement of Serum Biomarkers

Ath substantially (*p* < 0.001) augmented the serum levels of ALT, AST, VLDL, LDL, TC triglycerides, LDH activity, and AIP (atherogenic index in plasma) and lowered HDL level when juxtaposed to control treatment. Intraperitoneal injections (for 30 days daily) of CA 25 mg/kg exhibited marked decline in the plasma ALT (*p* < 0.001), AST (*p* < 0.01), VLDL (*p* < 0.001), LDL (*p* < 0.001), TC (*p* < 0.001), triglycerides (*p* < 0.001), LDH (*p* < 0.001) activity, AIP (*p* < 0.001), and enhancement in HDL (*p* < 0.001) relative to Ath control. Ath + CA 50 mg/kg and Ath + ATORVA (5 mg/kg) groups displayed a decrease in the serum ALT (*p* < 0.001), AST (*p* < 0.001), VLDL (*p* < 0.001), LDL (*p* < 0.001), TC (*p* < 0.001), triglycerides (*p* < 0.001), LDH (*p* < 0.01), and AIP (*p* < 0.001) and an increase in HDL (*p* < 0.001) content in relation with Ath control group (Table 1).

### 3.3. CA Improves Hemodynamic Parameters in Rats against Ath

Ath administration substantially (*p* < 0.001) amplified the SAP (systolic arterial pressure), HR (heart rate), DAP (diastolic arterial pressure), and decreased AP (arterial pressure) and MAP (mean arterial pressure) levels when juxtaposed to control treatment. Daily CA (25 mg/kg) treatments for 30 days significantly attenuated Ath caused upsurge in SAP (*p* < 0.05), HR (*p* < 0.001), DAP (*p* < 0.05) and also averted the reduction in AP (*p* < 0.05) and MAP (*p* < 0.05) in comparison with Ath control. The Ath + CA 50 mg/kg group exhibited a significant diminution in the levels of SAP (*p* < 0.001), HR (*p* < 0.001), and DAP (*p* < 0.01) and also displayed a significant upsurge in AP (*p* < 0.01) and MAP (*p* < 0.01) relative to the Ath control group. The standard group Ath + ATORVA (5 mg/kg) exhibited a significant diminution in the levels of SAP (*p* < 0.001), HR (*p* < 0.001), and DAP (*p* < 0.001) and also a significant growth in AP (*p* < 0.001) and MAP (*p* < 0.001) as compared to the Ath control group (Table 2).

### 3.4. CA Prevents Ath-Induced Increase in Oxidative Stress

Atherogenic diet instigated a significant (*p* < 0.001) intensification in the levels of thiobarbituric acid reactive substances (TBARS) and diminution (*p* < 0.001) in the levels of GSH in the whole heart when juxtaposed to vehicle treatment. CA (25 mg/kg, 50 mg/kg) or ATORVA (5 mg/kg) long-term treatment in Wistar rats significantly (*p* < 0.001) diminished the cardiac TBARS level against Ath in relation with rats that were given Ath and vehicle only. The Ath-induced decline in GSH levels was conspicuously averted by CA *i.p.* injections (25 mg/kg, *p* < 0.01; 5 mg/kg, *p* < 0.001) relative to rats treated with Ath and vehicle (Table 3). ATORVA (5 mg/kg)-treated rats also showed substantially (*p* < 0.001) enhanced GSH content relative to vehicle-treated Ath control rats. CA 50 mg/kg produced an antioxidative effect at par with the standard drug ATORVA against Ath diet in this study.

### 3.5. Effects of CA on the Staining of the Aorta

**Figure 3:**
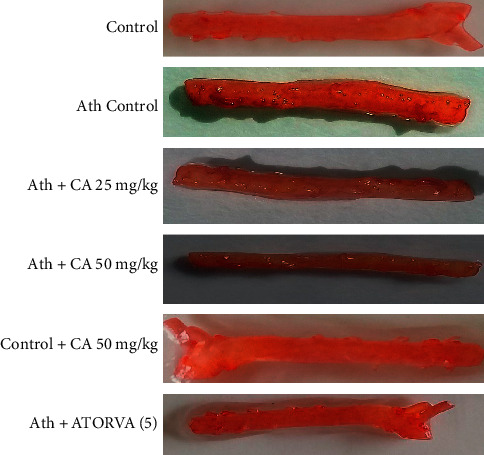
Caffeic acid (CA, 25 and 50 mg/kg) prevents aortic lesions in rats exposed to atherosclerogenic diet (Ath).

The aortic lesion percentage was found high in the Ath control group relative to normal chow diet groups (Figure 3). The aortic lesion percentage was decreased by atorvastatin (5 mg/kg) and moderately decreased by caffeic acid (25 and 50 mg/kg, *i.p.*) treatments relative to the Ath control group (Table 4).

## 4. Discussion

Approximately a century has witnessed considerable research and efforts in the field of cardiovascular disorders, particularly coronary heart disease biology and pathogenesis. Evidence substantiates the intricate involvement of dietary habits, tobacco, alcohol abuse, and physical inactivity in the initiation of coronary heart diseases [33]. Right from the initial stages, it is well recognized that higher consumption of dietary fats is an important inducer of coronary heart disease. Complex interactions between diet, lifestyle, and lipoprotein metabolism govern the progress of atherosclerosis and its associated complications. Various high-fat diet-induced experimental atherosclerosis animal models are available to assess the pathogenesis of atherosclerosis. A rise in body mass/weight and aggregation of fat is the leading pointers for the steady advancement of obesity [34]. Hyperlipidemia is the foremost hazard aspect for atherosclerosis that triggers inflammation and activates platelets and angiotensin-II leading to vascular smooth muscle proliferation and plaque formation. Epidemiological examination exposed a positive relationship between the degree of severity of atherosclerosis and the concentrations of blood cholesterol including LDL [35]. Ath augments lipid levels in the body and predisposes towards atherosclerosis [36].

In this study, supplementation of Ath resulted in a rise in the content of diverse lipids, namely, TC, LDL, VLDL, and triglycerides, in the bloodstream and a decline in HDL levels. Ath might damage endothelial cell function and integrity by augmenting free radicals and peroxidation of lipids, proteins, and genetic material. Estimation of biomarkers of oxidative insult disclosed that Ath supplementation caused an increase in MDA and a decrease in GSH in the heart homogenates. Atherosclerosis is a result of oxidative damage of the endothelial (or intimal) lining of vessels due to free radicals or lipid-free radical interaction toxins such as malondialdehyde or 4-hydroxy 2-nonenal [5]. An enhancement in the concentration of serum cholesterol and triglycerides of atherosclerosis rats may be a result of lipid peroxidation evoked by a high-fat diet [6]. GSH protects against oxidative damage by the removal of surplus free radicals and associated toxic adducts. GSH is an important tripeptide that acts as a major source of –SH (thiol) antioxidant [37]. Lipid peroxidation with pathogenic protein and DNA modifications helps immensely in the progression of atherosclerosis [21]. Experimental evidence substantiates that endothelial injury amplifies reactive oxygen species that trigger the peroxidation of cellular PUFAs (polyunsaturated fatty acids) that corroborates both functional losses of the myocardium and structural injury [8]. However, Ath-induced increase in TC, LDL, VLDL, and triglycerides in the bloodstream and a decline in HDL levels were attenuated by CA and the standard drug (ATORVA) in the existing study. Furthermore, AIP was decreased by CA and AORVA against Ath in rats. Data from previous studies also indicated that antioxidants impart reduction in atherosclerogenic factors [38]. In preclinical and clinical studies, data suggested that hypocholesterolemia agents can reduce clinical complications of atherosclerosis and prolong the life of a person by lowering the cardiovascular risk.

As a result of endothelial and intimal damage, cytosolic enzymes such as ALT and AST are released into the bloodstream with an increase in LDH activity and assist as the diagnostic indicators of myocardial tissue mutilation [39]. Ath is a well-known causative factor in myocardial tissue necrosis and heart dysfunctions marked by amplified end-diastolic volume, end-diastolic pressure, and left ventricular wall thickness. Long-term treatment with CA averted the escalation of ALT, AST, and LDH activities in the blood of animals that were given Ath for 30 days daily.

Triglycerides and lipids can be stained by Oil Red O that is a lysochrome (fat-soluble) diazo dye. This dye selectively stains fatty aggregates on the tissue surface [32]. Current experimental data suggest an 88.6% upsurge in aortic lesions in the Ath control group that was reduced to 36% in ATORVA (5 mg/kg)- and 15.3% and 27.2% in CA (25, 50 mg/kg)-treated groups, respectively. These findings indicate that CA has the potential to restrict the aortic lesions against Ath and this reduction in the aortic lesion is at par relative to ATORVA (standard drug).

## 5. Conclusions

The findings of the existing study indicate that caffeic acid (CA) might be used as an antiatherosclerogenic drug that lowers oxidative stress, ameliorate lipid levels, and reduce aortic injury. However, a deep insight is required to explore its exact antiatherosclerogenic mechanism by using different agonists and antagonists and molecular techniques to assess the role of signaling pathways. Furthermore, derivatives of CA can also enhance the bioavailability and efficacy against atherosclerosis. Further clinical investigations are required to substantiate the therapeutic use of CA in cardiovascular disorders such as atherosclerosis.

## Figures and Tables

**Table 1:** Effects of caffeic acid (CA) against atherogenic diet- (Ath-) induced alteration(s) in blood biochemistry.

Group	Cholesterol (mg/dL)	HDL (mg/dL)	LDL (mg/dL)	VLDL (mg/dL)	TG (mg/dL)	LDH (IU/L)	ALT (IU/L)	AST (IU/L)	AIP
Control	157.2 ± 1.515	65.17 ± 0.307	74.16 ± 0.477	30.17 ± 0.477	120.1 ± 0.167	130.1 ± 0.307	80.5 ± 0.428	9 ± 0.365	1.6 ± 1.141
Ath control	321.5 ± 2.861^a^	25.17 ± 0.307^a^	212.3 ± 0.615^a^	62.5 ± 0.563^a^	346 ± 0.365^a^	345.3 ± 0.307^a^	131 ± 0.365^a^	51.33 ± 0.365^a^	10.7 ± 0.23^a^
Ath + CA (25 mg/kg)	238.5 ± 3.442^z^	35.0 ± 0.3651^z^	139.8 ± 0.477^z^	42.5 ± 0.428^z^	250.83 ± 0.307^z^	236.5 ± 0.31^z^	119.16 ± 0.494^z^	31 ± 0.516^z^	5.4 ± 1.374^z^
Ath + CA (50 mg/kg)	187.2 ± 2.926^z^	48.5 ± 0.2236^z^	109.6 ± 0.558^z^	37 ± 0.447^z^	160.8 ± 0.401^z^	191.8 ± 0.308^z^	97.83 ± 0.365^z^	22 ± 0.364^z^	3.13 ± 1.137^z^
Control + CA (50 mg/kg)	157.8 ± 2.428	67.0 ± 0.333	70.8 ± 0.428	28.5 ± 0.429	119.6 ± 0.494	128 ± 0.365	78 ± 0.365	7 ± 0.966	1.4 ± 1.031
Ath + ATORVA (5)	131.7 ± 2.076^z^	48.0 ± 0.365^z^	92.5 ± 0.5^z^	33.6 ± 0.333^z^	131.1 ± 0.307^z^	161 ± 0.365^z^	88.3 ± 0.364^z^	18.1 ± 0.477^z^	2.5 ± 0.118^z^

Values are expressed as mean ± S.E.M. ^a^*p* < 0.001*vs.* the control group, ^z^*p* < 0.001*vs.* the Ath control group. Data are analyzed using one-way ANOVA followed by Tukey's HSD *post hoc* test. HDL : high-density lipoprotein, LDL : low-density lipoprotein, VLDL : very-low-density lipoprotein, TG : triglyceride, AIP : atherogenic index in plasma.

**Table 2:** Effects of caffeic acid (CA) against atherogenic diet- (Ath-) induced alteration(s) in hemodynamic parameters.

Group	AP (mmHg)	HR (BPM)	MAP (mmHg)	SAP (mmHg)	DAP (mmHg)
Control	120.1 ± 2.913	374.2 ± 8.171	120.06 ± 3.28	121.13 ± 4.248	85.5 ± 2.32
Ath control	96.33 ± 2.883^a^	449.2 ± 7.977^a^	93.36 ± 4.222^a^	144.15 ± 4.19^a^	99.83 ± 2.822^a^
Ath + CA (25 mg/kg)	104.8 ± 2.315^x^	403.8 ± 4.512^z^	110 ± 3.235^x^	134.3 ± 4.006^x^	97.5 ± 3.334^x^
Ath + CA (50 mg/kg)	112.8 ± 2.496^y^	386.7 ± 6.168^z^	114.7 ± 3.63^y^	129.5 ± 2.754^z^	95.17 ± 3.229^y^
Control + CA (50 mg/kg)	118.2 ± 5.029	372 ± 3.715	108.2 ± 4.672	121.3 ± 3.989	81.67 ± 1.054^z^
Ath + ATORVA (5)	114.67 ± 4.167^z^	380.2 ± 4.665^z^	119.2 ± 3.26^z^	125.3 ± 2.499^z^	83.17 ± 1.797^z^

Values are expressed as mean ± S.E.M. ^a^*p* < 0.001*vs.* the control group, ^x^*p* < 0.05, ^y^*p* < 0.01, and ^z^*p* < 0.001*vs.* the Ath control group. Data are analyzed using one-way ANOVA followed by Tukey's HSD *post hoc* test. AP : arterial pressure, BPM : beats per minute, HR : heart rate, MAP : mean arterial pressure, SAP : systolic arterial pressure, DAP : diastolic arterial pressure.

**Table 3:** Effects of caffeic acid against atherogenic diet- (Ath-) induced alteration(s) in oxidative stress markers.

Group	MDA (*μ*mol/ml)	GSH (nmol/ml)
Control	0.499 ± 0.003	0.468 ± 0.024
Ath control	0.859 ± 0.004^a^	0.133 ± 0.017^a^
Ath + CA (25 mg/kg)	0.738 ± 0.012^z^	0.229 ± 0.013^y^
Ath + CA (50 mg/kg)	0.62 ± 0.006^z^	0.387 ± 0.042^z^
Control + CA (50 mg/kg)	0.503 ± 0.0004	0.438 ± 0.02
Ath + ATORVA (5)	0.515 ± 0.002^z^	0.433 ± 0.004^z^

Values are expressed as mean ± S.E.M. ^a^*p* < 0.001*vs.* the control group, ^y^*p* < 0.01, ^z^*p* < 0.001*vs.* the Ath control group. Data are analyzed using one-way ANOVA followed by Tukey's HSD *post hoc* test. MDA : malondialdehyde, GSH : glutathione.

**Table 4:** Effect of caffeic acid on aortic lesions (%).

Group	Aortic lesion (%)
Control	1.34
Ath control	11.8
Ath + CA (25 mg/kg)	10.07
Ath + CA (50 mg/kg)	8.4
Control + CA (50 mg/kg)	1.3
Ath + ATORVA (5)	7.5

## Data Availability

Data of this study are available upon suitable request from the corresponding author.
